# Blockchain Platform For COVID-19 Vaccine Supply Management

**DOI:** 10.1109/OJCS.2021.3067450

**Published:** 2021-03-22

**Authors:** Claudia Antal, Tudor Cioara, Marcel Antal, Ionut Anghel

**Affiliations:** Computer Science Department, Faculty of Automation and Computer ScienceTechnical University of Cluj-Napoca 112961 400114 Cluj-Napoca Romania

**Keywords:** Blockchain, COVID-19, immunization programs, data integrity and immutability, smart contracts, vaccine distribution, transparency and audit

## Abstract

In the context of COVID-19 pandemic, the rapid roll-out of a vaccine and the implementation of a worldwide immunization campaign is critical, but its success will depend on the availability of an operational and transparent distribution chain that can be audited by all relevant stakeholders. In this paper, we discuss how blockchain technology can help in several aspects of COVID-19 vaccination scheme. We present a system in which blockchain technology is used to guaranty data integrity and immutability of beneficiary registration for vaccination, avoiding identity thefts and impersonations. Smart contracts are defined to monitor and track the proper vaccine distribution conditions against the safe handling rules defined by vaccine producers enabling the awareness of all network peers. For vaccine administration, a transparent and tamper-proof solution for side effects self-reporting is provided considering beneficiary and administrated vaccine association. A prototype was implemented using the Ethereum test network, Ropsten, considering the COVID-19 vaccine distribution conditions. The results obtained for each on-chain operation can be checked and validated on the Etherscan. In terms of throughput and scalability, the proposed blockchain system shows promising results while the estimated cost in terms of gas for vaccination scenario based on real data remains within reasonable limits.

## I. Introduction

Covid-19 virus part of the coronavirus ribonucleic acid virus family [1] has generated a worldwide pandemic being very easy to spread and pushing a lot of pressure on the healthcare system and levels of the society. Since its identification in Wuhan, China in December 2019, it has spread rapidly through community transmission generating up to December 2020 to around 65 million confirmed cases and more than 1.5 million deaths [2], [3]. Even if significant efforts have been made for fighting the pandemic, the spreading rate of the virus was only slowed. In many countries, the restriction measures are still in place to avoid suffocating the hospitals and treatment centers [4].

In this context, the rapid roll-out of a vaccine and the implementation of a worldwide immunization campaign is critical for the control of the pandemic. Since the beginning of the pandemic, the pharmaceutical companies have concentrated their efforts on developing a vaccine in record time to achieve COVID-19 containment [5], [6]. While some COVID-19 vaccines are in the final test phases, preparing and planning for mass immunization becomes extremely important. Nevertheless, several aspects are likely to influence the success of the COVID-19 immunization program. In our opinion blockchain, may provide the technological means for addressing them.

The first aspect is the availability of an operational and transparent end-to-end supply chain and logistics systems [7], [8]. Its role is to assure vaccine storage and stock management, and rigorous temperature control in the cold chain [5]. Table 1 shows the storage and distribution requirements of the most likely vaccine candidates.

**TABLE 1:** COVID-19 Candidate Vaccines Temperature Control Conditions [10], [11]

	**Freezer temperature**	**Refrigerator temperature**	**Max storage days**
Pfizer	- 70 degrees Celsius	N/A	30 days after opening the freezer
Modena	- 20 degrees Celsius	2-8 degrees Celsius	30 days in the refrigerator
Oxford-AstraZeneca	N/A	2-8 degrees Celsius	6 months in the refrigerator

Blockchain can increase the efficiency and transparency of COVID-19 vaccine distribution assuring the traceability and the rigorous audit of the storage and delivery conditions. Blockchain-based solutions may provide a fully automated implementation of data accountability and provenance tracking in vaccine distribution. In this way, it will enable the integration of different information silos owned and managed by different types of stakeholders on the entire distribution chain. Self-enforcing smart contracts may assure the traceability of the COVID-19 vaccine supply chain. This is important in the cold part of the chain, where the vaccine needs to be kept at extremely low temperatures to remain viable. A breach in guaranteeing the delivery conditions will be registered on the blockchain in a tamper-proof manner. All network peers will be made aware due to the distributed ledger block distribution and replication features. Finally, the blockchain can act as proof of the delivery chain, making it very difficult to counterfeit the vaccine. At any point, the medical units and the vaccine beneficiaries would cab trace it back up to the companies that have registered the vaccine lots in circulation.

The second aspect is the transparency and correctness in the registration and management of the waiting list of people for immunization. The data on this list is not only sensitive but at the same time, it requires correctness, avoidance of impersonation, privacy, and immutability. These properties can be provided by using blockchain technology. Blockchain can change how the waiting list is managed by allowing parties mutually unknown to transact the vaccine as a digital asset securely without a central trusted intermediary. Such a decentralized system will remove the necessity of having third parties’ entities that centralize and manage the waiting list. The immutability of transactions and the authorization provided by using smart contracts allow all network peers to restrict access to their private information. All actions executed by a smart contract may be propagated across the network and recorded on the blockchain, and therefore are publicly visible. Transactional privacy, as well as the privacy of personal data, can be assured using novel solutions such as the incorporation of zero-knowledge proofs which are cryptographic techniques that can enforce privacy for verifying private data without revealing it in its form [12], [13].

Finally, the third aspect is building trust in the vaccine effectiveness by implementing a transparent and public reporting system of potential side effects. It includes the automatic tracing back up vaccine lot level and mapping of reported side effects. Concerns have been raised that different drug makers do not report correctly and completely the side effects to relevant authorities [14], [15]. Thus, a transparent and reliable system to report the side effects once a drug/vaccine is released is crucial. In this sense, a blockchain platform would bring advantages concerning the existing state of the art solutions. Any beneficiary that has received a vaccine, will report any problems/symptoms encountered after the administration using blockchain. A transaction will be stored associating the vaccine lot and will be replicated in the network. All other peers will be made aware and the report could be validated using the peers’ consensus concerning vaccine lot. Furthermore, being stored in an immutable log, all the reported side effects are protected against tampering.

Analyzing the existing state of the art literature reviewed in Section II one may see that there are many applications of blockchain that are investigated such as contact tracing, immunity passport, and COVID-19 diagnosis [51]. Even though blocking technology has some relevant features in addressing all the three critical aspects for implementing a successful immunization campaign, very few approaches can be found in the literature. Moreover, most of the blockchain solutions proposed to manage the COVID-19 vaccine supply chain are in the early phase of design studies [27], [37], or are viewpoints from blockchain companies or stakeholders [41], [46], [47].

In this paper we introduce a blockchain-based system for the transparent tracing of COVID-19 vaccine registration, storage and delivery, and side effects self-reporting. Leveraging on blockchain technology features it describes the development of the following novel mechanisms:

• A blockchain-based solution for data immutability, transparency, and correctness of beneficiary registration for vaccination to avoid the problem of identity thefts and impersonations.
• A decentralized smart contracts-based solution for monitoring the proper vaccine transportation conditions in a cold chain and real-time awareness of all peers about the fulfillment of COVID-19 vaccine delivery and storage conditions.
• Smart contracts-based solution for vaccine administration and tamper-proof self-reporting of side effects, person identification, and vaccine association.

The rest of the paper is organized as follows: Section II describes the relevant related work in the area of Information and Communication Technology (ICT) and blockchain solutions for managing immunization campaigns; Section III presents the methods and procedures for the proposed blockchain system for safe vaccine distribution and Section IV presents a test case for COVID-19 vaccine distribution tracking and system scalability results. Finally, Section V presents conclusions and future work.

## II. Related Work

ICT solutions for supporting immunization campaigns are proposed in the state of the art mostly for optimal distribution planning of vaccines [16]–[18]. In [19] a drive-through vaccination simulation tool is proposed for planning and feasibility assessment of such facilities based on event processing and agent-based modeling to minimize waiting times, staff required, immunization intervals, etc. Vaccine distribution for heterogeneous population has been approached by using mathematical modelling using equity constraint to maintain fairness and to optimize the number of vaccine doses in case of an influenza outbreak [17], [18]. Various heuristics and custom optimization algorithms are proposed for optimizing the distribution network design [20]–[22].

Recent advancements of contemporary technologies such as Internet of Things (IoT), machine learning and blockchain pave the way for building more smart and innovative systems that can be adapted to different domains as it is the case of the healthcare domain [23]. The authors of [24] propose the use of IoT devices to monitor the location of the carrier, temperature and humidity with the goal of optimizing and increasing vaccine coverage in the remote regions and ensuring transparency in the overall process. Blockchain based decentralized systems for addressing healthcare sector problems such as privacy and confidentiality of data are presented in [25], [26]. Recent studies have pointed the possibilities of using blockchain in combating the COVID-19 pandemic most of them addressing the decentralized tracking of contracts and symptoms or for assuring security and immutability [27]. Relevant use cases for blockchain technology in managing COVID-19 pandemic contact tracing, patient data sharing, supply chain management are overviewed in [28]. Other studies have shown that blockchain can be used to develop trustful predictive systems that can help containing the pandemic risks on national territory [29] or to securely track the movements of residents in quarantine scenarios using IoT infrastructures [30]. Incentive based approaches have been proposed to battle against the COVID-19 pandemic that use blockchain to prevent information tampering and incentives for rewarding patients to remain in quarantine [31].

Blockchain has been proposed as a solution for organization and management of industry supply chains [32]. For pharmaceutical supply chain where temperature monitoring or counterfeit drug prevention are of utmost importance, IoT and blockchain frameworks may offer a viable solution [33]. In [34] a blockchain drug supply chain management is combined with a machine learning recommendation system. The supply chain management system is deployed using Hyperledger fabrics to continuously monitor and track the drug delivery process while N-gram, LightGBM models are used to recommend the best medicines to the customers. In [35] Gcoin blockchain is proposed for the data flow of drugs to create transparent drug transaction data where every unit that is involved in the drug supply chain can participate in the same time to prevent counterfeit of drugs.

Few approaches in the literature are addressing the development of blockchain based systems for distribution of vaccines. Authors of [36] propose a blockchain management system for the supervision of vaccine supply chains through smart contracts also dealing with vaccine expiration and fraud recording. Machine learning models are used for recommendations to choose better immunization methods and vaccines. A blockchain model for the COVID-19 vaccine distribution chain is proposed in [37]. The approach proposes monitoring each phase from development to application, considering the emerging and commercial chains while leveraging on blockchain for authenticating each process and registering changes. Similarly, in [38] efficient supply chain management through smart containers with IoT sensors is proposed and used to administer shipment, payments, legitimize receiver, etc. VeChain [39] is developing a blockchain-based platform for vaccine production and tracing in China using IoT devices to capture vaccine production data and store it an enterprise blockchain to ensure immutability. Finally, blockchain can help to track the vaccines and make sure they haven't been compromised or even to keep track of patients’ vaccine records and provide proof of vaccination especially because COVID-19 will require two vaccine doses [40], [41].

## III. Blockchain and Smart Contracts

Blockchain provides the technological infrastructure for developing decentralized applications [52]. It features a linked list of blocks chained using hash pointers, each block storing transactions for an asset that may be digitized [53]. Thus, at its core, blockchain technology relies on hash-based data structures that provide benefits like collision-free, data binding, and data concealing. As a result, the transactions are stored in tamper-evident data structures, creating a log of state transitions offering data provenance and traceability capabilities. The state transitions can refer either to a coin transaction like in Bitcoin [54], to smart contracts state updates such as Ethereum [55], or to a property as in DigiShares [56], etc.

Mining algorithms seal the log of transactions by the agreement of all network participants, making it immutable. The immutability of the structures combined with the security offered by asymmetric cryptography makes blockchain a powerful technology in building safe and reliable decentralized systems. Anyway, the attacks of malicious participants need to be addressed. They are joining the network to disrupt the correctness and integrity of the validation process or to create forks in the linear structure of the chain. This is avoided in blockchain due to the complexity of the mining (e.g., Proof-of-Work algorithm [57]) or other type consensus algorithms [43] [58] requiring the agreement of over 50% of network participants. The most used consensus algorithms in the blockchain networks are the Proof Protocols. Proof of Work (PoW) is the consensus algorithm used by two of the most known public blockchains, Bitcoin and Ethereum. It requires for a block to be considered valid, to contain the solution of a computationally intensive puzzle. In the mining process, the participant node that manages to solve the puzzle will append the block with the most recent transactions to the chain. Thus, a malicious participant cannot influence the validation decisions of the other nodes, if it does not own most of the mining power in the network. The amount of computing power needed for a successful attack will continue to increase with the number of trustful nodes in the network thus becoming unlikely.

The introduction of smart contracts, as stateful pieces of code executed by all network participants changed how the applications are implemented. Smart contracts can programmatically impose business rules regarding the transfer of assets. A new development paradigm has emerged for decentralized applications that are governed by the same features as the transactions of cryptocurrency. The smart contracts code, once written, is immutable and subject to the consensus algorithm such as the sealing of transactions. This facilitates the implementation of decentralized applications in which the business logic could now be written in smart contracts using different Turing Complete programming languages and are deployed on blockchain networks offering highly secured solutions.

Finally, the QR codes [59] are 2D matrix barcodes that can store a lot of information about the physical asset with which they are associated. They may be processed by all popular smartphones, making them a convenient to assure the link to digital assets and systems. Recently QR codes have been adopted and integrated with blockchain solutions for decentralized tracking of various assets [60], [61]. Featuring automatic generation and scanning, they increase the processes reliability especially on the human interaction part. Also, in the COVID 19 pandemic QR codes became the main method touchless interaction especially in hospitals [62], [63].

Considering the state of the art reported in Section II and the presented technological background and advantages, we propose a blockchain system that manages the distribution and administration of COVID-19 vaccines. In our case, the main considered digital asset is the vaccine. Transactions describing the interactions of various actors with the system and the progression of vaccines through the supply chain from creation until administration are registered on the blockchain. Smart contracts implement the vaccine distribution administration rules being replicated in each node of the network allowing for transaction validation.

Transactions are aggregated in a block which is replicated in the network allowing each node to validate the state transitions. The proposed changed state is accepted and inserted in the chain only if its validation is successful, otherwise, the block is dropped. Each transaction is tracked and validated by each node locally, before unanimously accepting it into the history. In this way, we offer a replicated and highly reliable solution because each node will validate the vaccine and beneficiary registration, vaccine distribution and administration, etc.

Moreover, to automatize the integration with the physical world, QR codes are integrated with the blockchain system. In our case, a QR code provides information regarding the vaccine registration, a 34 character hash representing the smart contract address, the registration proof, a hash of alphanumeric characters identifying a transaction that has been mined in the blockchain, the vaccine lot id, etc. Furthermore, since all this information is immutably stored in the blockchain system, the associated QR code once generated remains valid.

Finally, our system works on a public blockchain such as the Ethereum public chain configuration. To conduct a successful attack, the malicious nodes need to have more than 50% percent of the computational power of the Ethereum network miners which is extremely unlikely.

## IV. Methods and Procedures

The traditional COVID-19 vaccine scheme [48] is considered fragile mainly due to the challenges imposed by the vaccine distribution, tracking, and registration. Several issues need to be carefully monitored and audited: vaccine damage due to its transport conditions; lack of coordination for distribution and registration; shortage of personnel; limited capacity [49]. In this distribution scheme, the containers must move across the world while IoT devices and sensors need to keep track of their temperature and location and this data should be stored and validated for assuring that vaccines are not corrupt. The vaccine producers need to prepare lots of vaccines and provide the associated data for their fair and trustful distribution. The medical centers will receive the vaccines and will need to make sound planning for their administration. Also, they will need to find solutions to avoid impersonations. The beneficiary must have safe and secure access to the vaccine and have the possibility to report side effects in a trustful manner. All these processes currently involve human intervention and manual activities that cannot assure a high level of security and privacy and finally will led to delays and prolong the pandemic [50].

**Figure 1.**
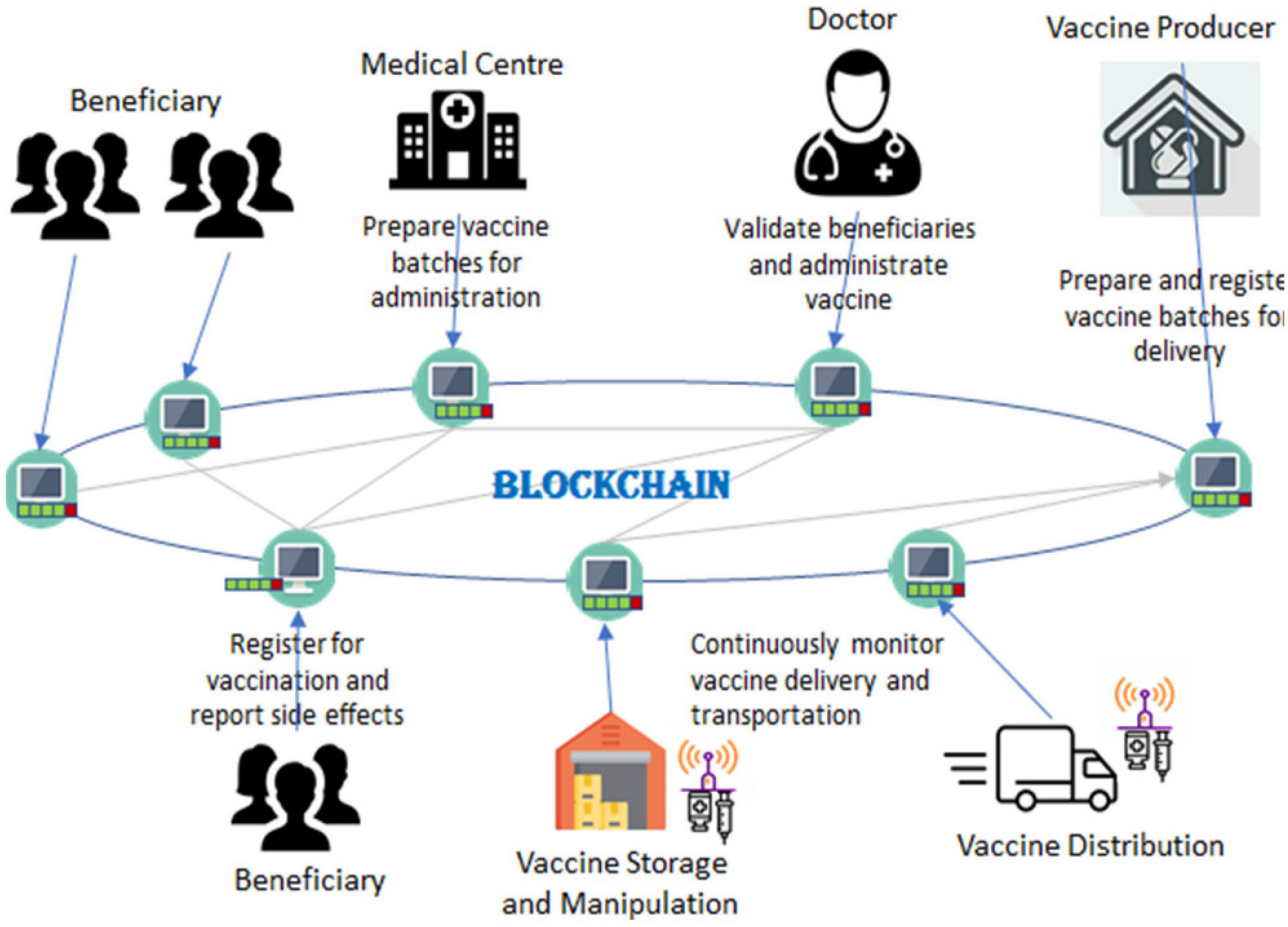
Blockchain and immunization program management: vaccine registration, tracking, monitoring, administration, and self-reporting.

To digitize and decentralize the traditional scheme, we propose a blockchain system which allows for transparent COVID-19 vaccine tracking, distribution monitoring, and administration (see Fig. 1). It uses the distributed ledger for storing vaccine data assuring information immutability. It provides reliable information related to the vaccines safe transportation to the beneficiaries, and for the identification other the of main actors involved in the vaccination scheme.

The main actors of the traditional vaccination scheme will act as peer nodes in the proposed blockchain-based system: i) the beneficiaries that register for vaccination, ii) the company that prepares and registers the vaccine batches/lots for transportation, iii) the IoT sensor devices that continuously monitor the vaccine delivery, storage and handling; iv) the medical centers that will receive the vaccine and prepare it for administration and v) the doctor who validates the beneficiary, delivery and storage conditions and administers the vaccine. All the actions are registered into the distributed ledger as immutable transactions which are stored in blocks that are replicated to all the peer actors in the chain. This will provide high transparency of the vaccine handling operations enabling the tracking and registration of the COVID-19 vaccine as a digital asset.

The main features of the system as well as their implementation using self-enforcing smart contracts are detailed in the next sub-sections.

### A. Immutable Registration of Vaccine Beneficiaries

**Figure 2.**
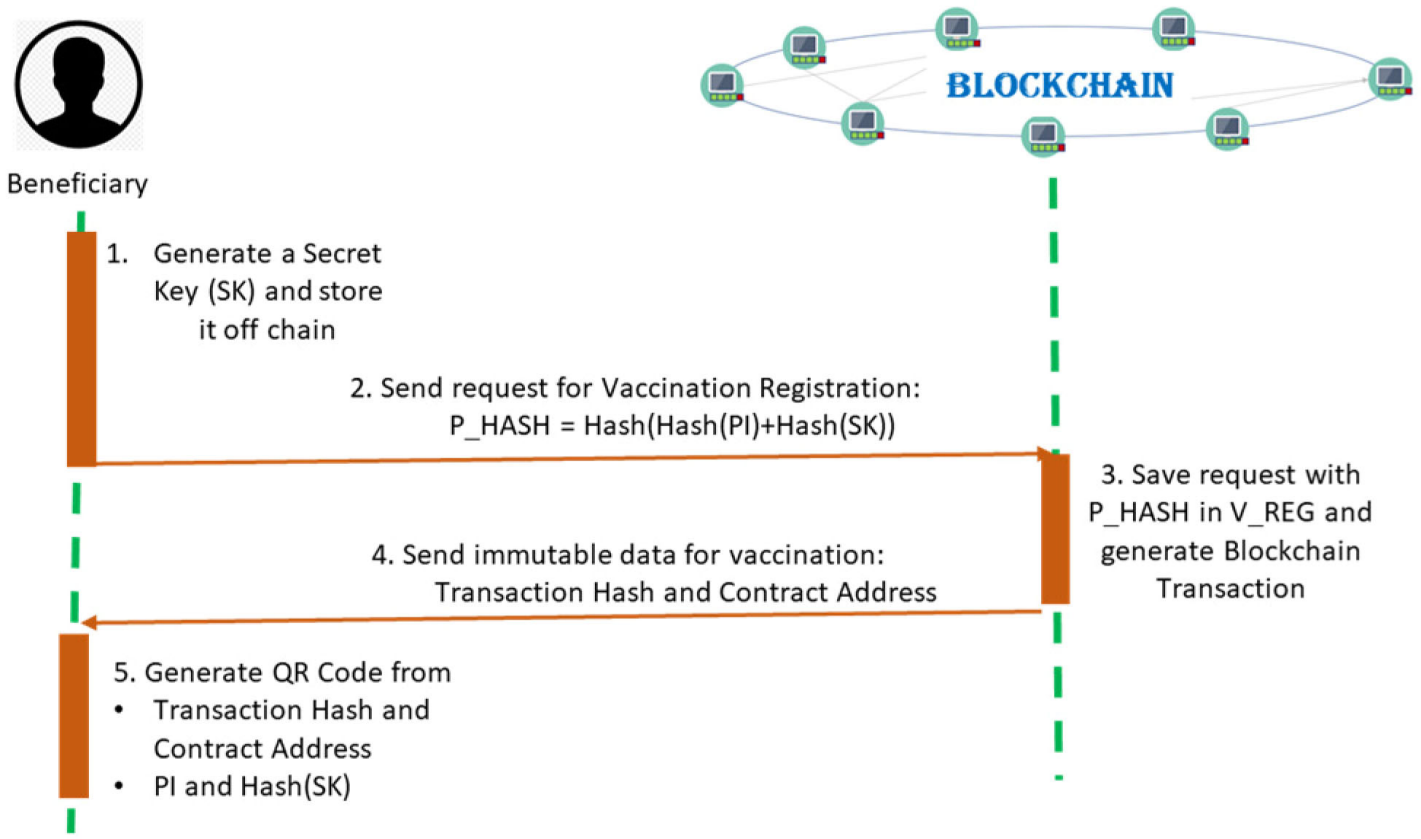
Immutable registration of vaccine beneficiary with the blockchain system.

Beneficiary registration on the blockchain-based system ensures the privacy of identity and avoids impersonations. As shown in Fig. 2, before the registration, the beneficiary generates a secret key (SK) that will be stored off-chain and will be later used to prove his/her identity. A Merkle Proof [42] is used to maintain personal data privacy and anonymity on-chain, while at the same time enabling the beneficiary to prove their identity without revealing it. A hash of the SK is generated for the beneficiary and stored in the root node together with the hash of the Personal Identification Number (PI) from the identity card. A hashing algorithm is used for automatically generating the hash of the PI and SK.

\begin{equation*}
P\_HASH\ = \ Hash\left({Hash\left({PI} \right),\ Hash\left({SK} \right)} \right)\tag{1}
\end{equation*}

The root of the Merkle Proof (P_HASH) becomes the payload of a blockchain transaction signed by the beneficiary and aimed for the Vaccine Registry (V_REG) contract deployed on chain, marking the intent to receive the vaccine.

Once mined, the transaction hash and the contract address are sent back to the beneficiary. A QR Code is automatically generated for the beneficiary, storing: the transaction hash, the contract address, the PI, and the hash of the SK. It will that will be later shown by the beneficiary to the doctor for its reliable identification. The QR code uses standardized encoding and decoding (Reed–Solomon error correction [63]) for data and will allow to easily identify, verify, and validate the beneficiary when administering the vaccine by simply scanning it in the system.

**Figure 3.**
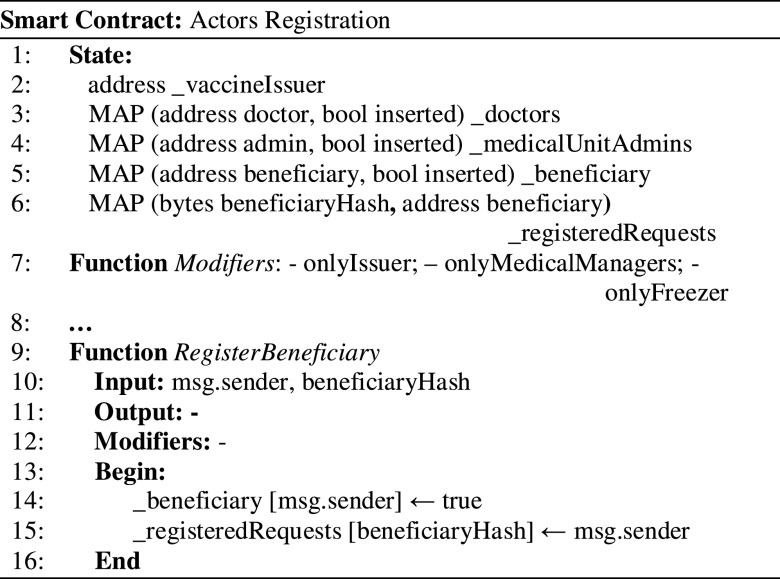
Registering vaccination scheme actors’ actions on the chain.

All relevant actors’ registration actions are managed using smart contract functions (see Fig. 3). We have used a map structure to keep track of the registered actors because it features an acceptable time overhead to access the resources for verification and validation. This leads to lower execution costs of the blockchain transactions. The beneficiary can register the request and intent for receiving the vaccine, and the address signing the registration transaction will be stored. As input payload, the beneficiary must provide the *beneficiaryHash* (line 10) represented by the Merkle Root.

During the verification step conducted by the doctor, the beneficiary will need to reveal the raw data. In this way, she/he will prove that he/she is the actual person who made the registration request using a pseudo-anonymized blockchain address. Upon smart contract deployment, the vaccine issuer address is stored as the one that has signed the deployment transaction (line 2). The functions for action registration associated with other actors such as Medical Centers, vaccine producers, or IoT devices ensure that their transactions are signed with corresponding verified addresses (line 7).

### B. Vaccine Distribution Chain Monitoring

**Figure 4.**
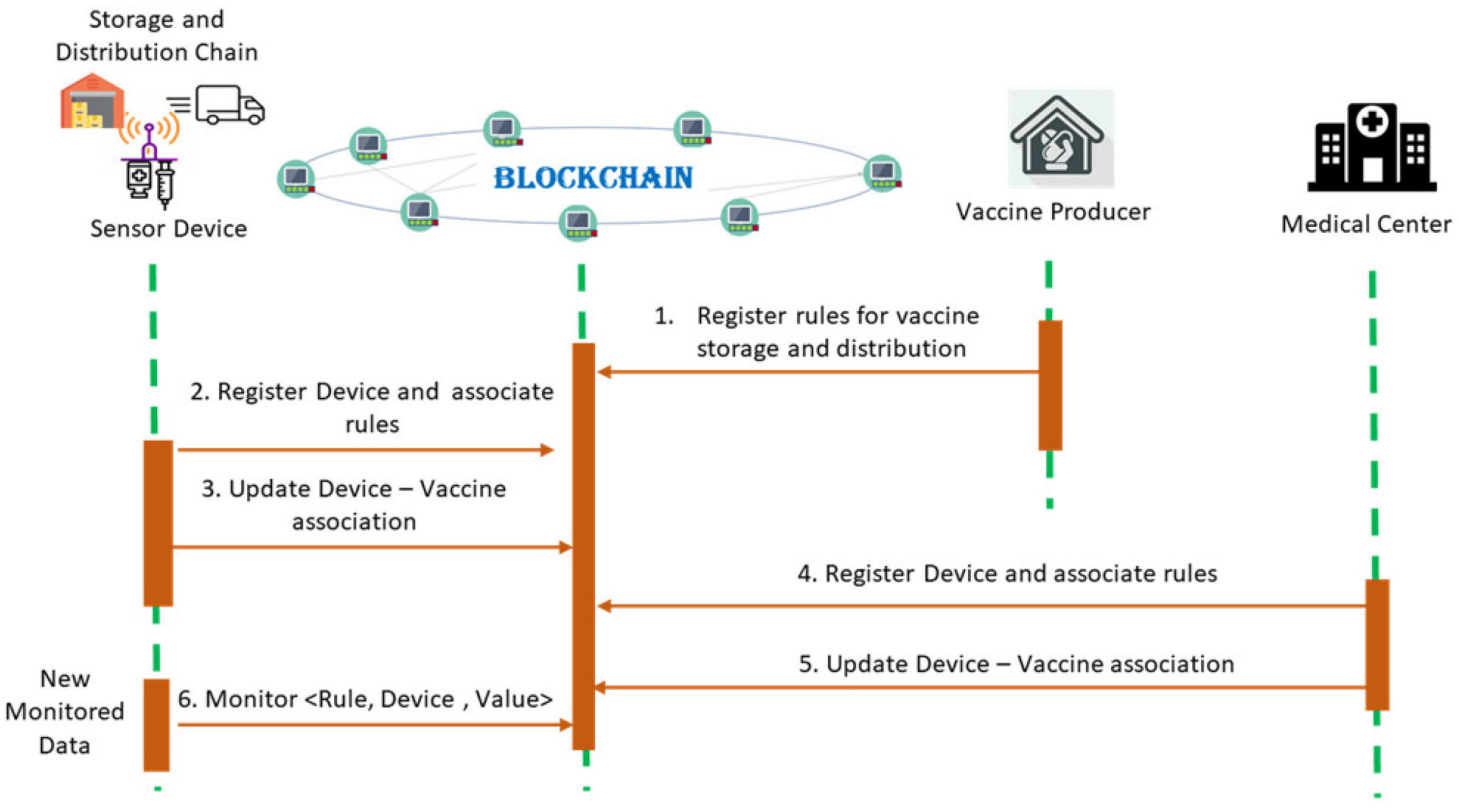
Monitoring the vaccine distribution chain.

The goal, in this case, is to make transparent the degree to which the defined conditions for vaccine storage and manipulation are met during the entire distribution chain. Smart contracts are used to evaluate continuously the data received from sensors deployed on storage units or attached to the transportation freezers against the defined conditions rules (see Fig. 4).

First, the vaccine producer company must register a set of rules for safe distribution and storage of the vaccine batches (see Fig. 4). The rules are encoded in smart contracts associated with specific IoT devices as rules that must be checked each time new data is provided:

\begin{align*}
Rul{e_k}:\ Mi{n_{Value}} <& Monitore{d_{Value}}\left(t \right) < Ma{x_{Value}},\\
\forall k \in& \left\{ {1..R} \right\}\ \text{and}\ t < TIM{E_{LIMIT}}\tag{2}
\end{align*}

The companies in the distribution chain or the medical centers can register a set of freezing devices used for vaccine manipulation and storage. The vaccine lots can be transported and stored in different types of devices depending on the destination up to distance and travel time (Fig. 4). At the same time, they may update the correlations of the freezing devices with vaccine lot. This is done by mapping the vaccine batch ready to be transported to a freezer ID that has associated a set of rules. All the associations and rules are stored in the blockchain distributed ledger making them difficult to be modified.

The IoT devices are responsible to sign blockchain transactions containing the monitored data. The transactions must contain the following payload:

\begin{equation*}
T{r_{payload}} = \ \left\langle {V{L_{ID}},Rul{e_k},Monitore{d_{Value}}\ \left(t \right)} \right\rangle \\tag{3}
\end{equation*}

specifying the vaccine batch identifier $V{L_{ID}}$, the value monitored by the sensor and the rule $Rul{e_k}$. Once the transaction is mined, the smart contract will verify the identity of the device that has signed the transaction and the monitored value against the rule limits defined for safe vaccine handling. After the transaction is stored in the blockchain, it triggers the execution of the smart contract rules. The results of execution are the validation or invalidation of the vaccine transportation in safe conditions. Using transaction time registered on the blockchain, the rule specifying the maximum time limit for transportation and storage is validated.

By storing the monitored values and the rules on the blockchain, the immutability and integrity of the data are assured. The monitored values cannot be changed and the decision of annotating these values as corresponding/breaking the issuer-imposed rules are subject to consensus and mined in the chain in a tamper-resistant manner. Any actor may check the registered logs and trust that the results provided were not subject to malicious tampering.

**Figure 5.**
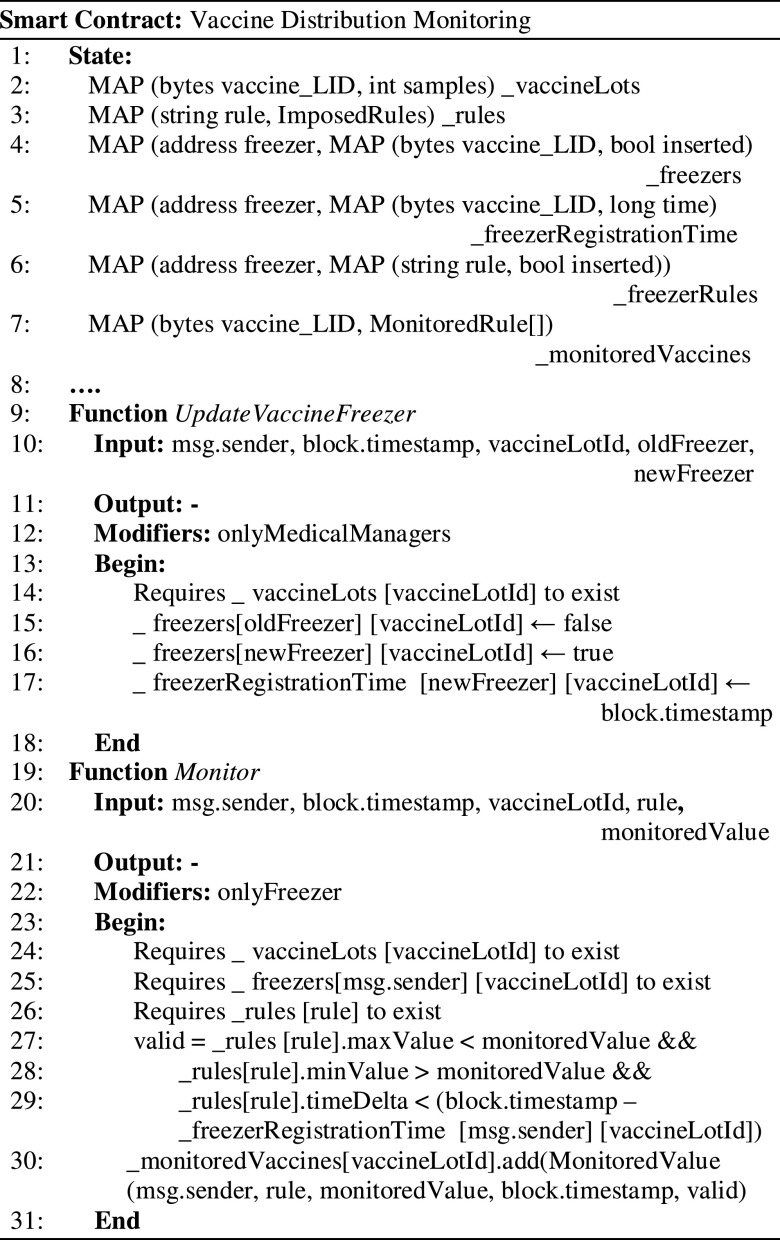
Monitoring and tacking the vaccine over the distribution chain.

Fig. 5 presents the smart contract used to track the vaccine distribution against the defined handling and storage rules. The freezer devices and vaccine lots are registered on the chain. The vaccine lots are assigned to freezing devices, this association being updated during the distribution chain enacting its decentralized tracing (see lines 9-18). Each time a monitored value associated with a freezing device is provided it is checked against the imposed rules (line 26-29).

The data regarding the monitored vaccine lot, all the information regarding the validity of the rules, and the time of transactions registration are stored in the blockchain in a tamper-proof log managed by the contract (line 30-31).

### C. Vaccine Administration and Side Effects Reporting

The most complex operation of the pipeline is the actual vaccine administration. This step must check and validate the following conditions for the blockchain system operation: the identity of the beneficiary to be vaccinated, the conditions of vaccine delivery and handling according to the rules defined by the producer, and the association of beneficiary with the vaccine to be administrated enabling the further reporting of potential side effects.

**Figure 6.**
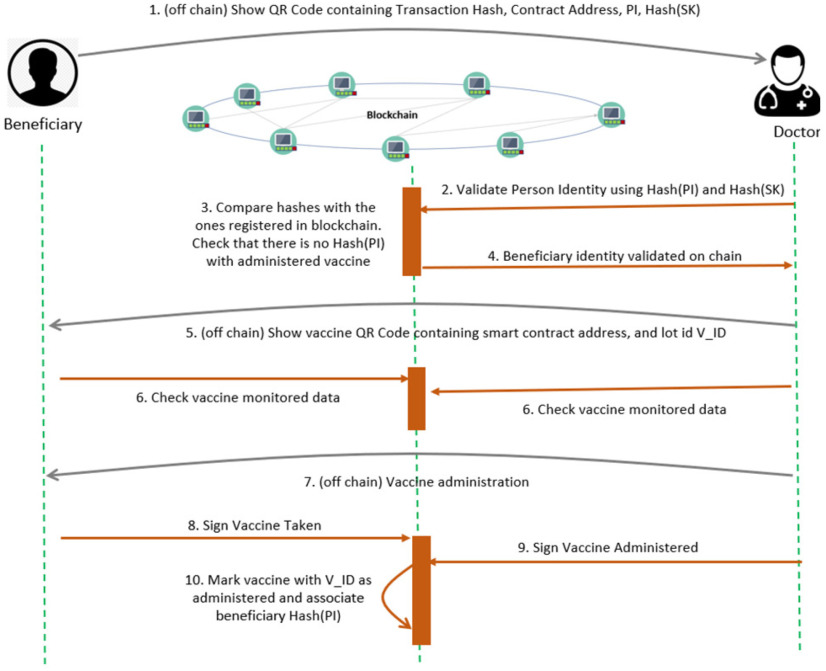
Vaccine Administration Sequence Diagram.

The first step of the process is the validation of beneficiary identity before the vaccination (see Fig. 6). It is performed by the doctor using the beneficiary registration QR code which contains the blockchain transaction hash, the smart contract address, the hash of personal identification number, and the hash of the secret key. Using the hashes extracted from the QR code, the doctor performs an on-chain identity verification for registration acknowledgment. Using the Merkle Proof, the hash of the two values is compared against the root stored in the blockchain during the beneficiary registration step.

After the beneficiary identity verification is completed, the vaccine QR Code is scanned to extract relevant vaccine information stored on the blockchain, such as the smart contract address that had registered it, the vaccine lot ID, and transportation conditions.

**Figure 7.**
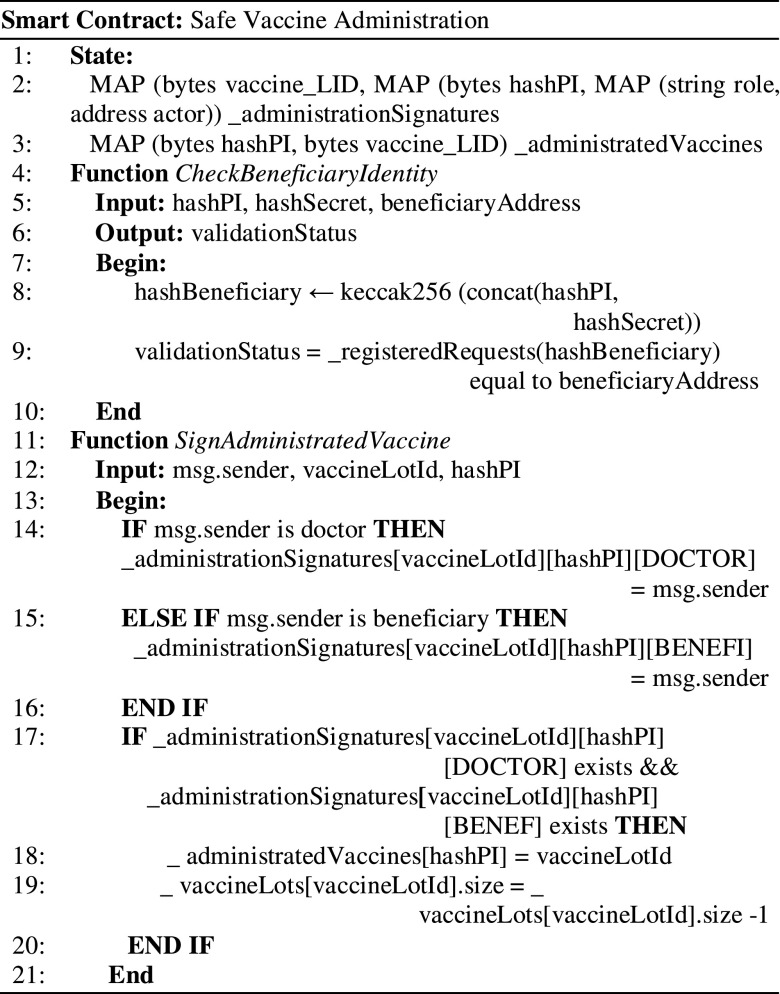
Validating the beneficiary and vaccine transportation and storage conditions.

After the vaccine is administrated, a two-step locking mechanism is employed to mark the vaccine on the blockchain using the signatures of the doctor and the beneficiary. In the blockchain, the vaccine is marked as administrated using the Hash (PI) of the beneficiary that had received the vaccine. The smart contract managing this process is presented in Fig. 7.

First, the beneficiary validation using QR code information and blockchain registered data is done (lines 4-10). This is done on the chain by offering as input the two hashes (the hash of the PI, and the hash of the SK) and checking the obtained root hash against the chain stored beneficiary registry. Both the beneficiary and the doctor must acknowledge the administration on the chain (lines 11-22) by signing the associated blockchain transaction. When both signatures are registered, the vaccine lot size is decremented and the association between the beneficiary Hash (PI) and the vaccine lot is registered (lines 19-20).

**Figure 8.**
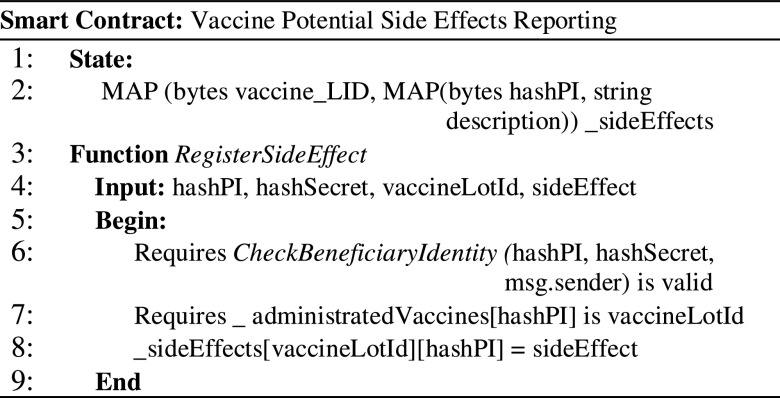
Smart contract for side effect reporting.

Any beneficiary that has received a vaccine can register feedback and the eventual side effects encountered (see Fig. 8). By registering the side effects directly on the chain, the information is difficult to be changed and censored. The beneficiary will sign a blockchain transaction that is authenticated and authorized (line 6), verified against the vaccine administrated from the specified lot (line 7), and correlated with reports of the other beneficiaries. As result, the side effect registered is stored on-chain as a transaction (line 8). Once the side effect is registered by the beneficiary, the information is stored as an immutable log, thus any attempt of third parties to alter it will be unsuccessful.

## V. Results

To evaluate our blockchain-based system we considered a setup for the COVID-19 vaccine using the rules for safe transportation and storage presented in [10]. We have considered that the distribution company delivers the vaccine lots to the interested medical care unit. It uses transportation freezers that must reach their destination in a maximum of 10 days. The vaccine should be kept all this time at a temperature between -80 and -60 Celsius degrees. Once reaching the destination, the medical unit will transfer the vaccine lots into the storage freezers where the vaccines can be deposited up to 5 days at a temperature between 2-8 degrees Celsius.

### A. COVID-19 Vaccine Distribution Tracking

A prototype has been tested on a public Ethereum test network, Ropsten [44]. The results obtained for each on-chain operation can be validated on the Etherscan [44] considering the transaction hash value reported in the next sub-sections. Etherscan was chosen because is one of the most important services for analyzing real-time Ethereum network statistics and blockchain simulations. Also, it is open, with no registration, and fees are needed for a third party to check our system results. We are describing system operations executed on-chain and the associated blockchain transaction receipts. Information related to the actor who signs the transaction, the executed call, and the transaction costs in gas consumed are highlighted. For consensus, we have used the PoW algorithm on the default configuration of the public chain [57].

*Actors and rules registration in the immunization program*

We start by deploying the smart contract for registering the main actors of the vaccination campaign on the blockchain (Table 2).

**TABLE 2:** Blockchain Transactions for Smart Contract Deployment

**Operation**	**Contract Deployment**
Signing Address	0xD2796dE988975DD292e8aC981c4011B23E801DCd *(Vaccine Issuer)*
Transaction Receipt	**transaction hash:** 0xaed1e3267afa17f22d5a125cda449945407c2485b299a8a15d9c0ff2beec6738
	**from:** 0xD2796dE988975DD292e8aC981c4011B23E801DCd
	**to:** VaccineRegistry.(constructor)
	**transaction cost:** 2327309 gas
	**input:** 0x608...40033

The vaccine producer must use its own Ethereum address to sign the transaction that triggers the smart contract deployment.

Next, the vaccine producer must sign transactions for registering the recipient medical care units as receivers of the future vaccine lots. For this, a medical unit administrator must be registered on the blockchain. Similarly, the doctors that will administer the vaccines are registered (see Table 3).

**TABLE 3:** Blockchain Transactions for Registering Immunization Camping Actors in This Case a Doctor

**Operation**	**Register Doctors**
Signing Address	0xD2796dE988975DD292e8aC981c4011B23E801DCd *(Vaccine Issuer)*
Transaction Receipt	**transaction hash:** 0x563ddcd6fba93dbb3c05d6cb6b366a9289efcf8dd209d5516dbee66e86a8bbc6
	**from:** 0xD2796dE988975DD292e8aC981c4011B23E801DCd
	**to:** VaccineRegistry.registerDoctor(address) 0x536798D9D1f0507C1a8600d9A475d410a90D5A0A
	**transaction cost :** 43798 gas
	**input:** 0x699...26efa
	**decoded input** { "address doctor": "0xF3A1846C82c74EA5D5d32a9BB8759A8093C26eFa" }
	**input:** 0x608...40033

The vaccine producer must register the rules for safe transportation and storage of the vaccines. In the depicted scenario two transactions must be registered: one setting the rules for transportation and one configuring the rules for storage (see Table 4).

**TABLE 4:** Blockchain Transactions for Registering the Vaccine Safe Distribution Rules

**Operation**	**Register Tracking Rules**
Signing Address	0xD2796dE988975DD292e8aC981c4011B23E801DCd *(Vaccine Issuer)*
Rule	The vaccine should be transported at a temperature between -80 and -60 Celsius degrees for maximum 10 days.
Transaction Receipt **(Transportation Rule)**	**transaction hash:** 0xbc8b46345096d4a69faccd7300cf3450f1fc66d1e238236d511dce1771aacf9f **from:** 0xD2796dE988975DD292e8aC981c4011B23E801DCd **to:** VaccineRegistry.registerTrackingRules(string, int32, int32, uint256) 0x536798D9D1f0507C1a8600d9A475d410a90D5A0A **transaction cost :** 216219 gas **input:** 0x4a2...00000 **decoded input** { "string rule": "transport-temperature", "int32 minValue": -80, "int32 maxValue": -60, "uint256 timeDelta": {"type": "BigNumber", "hex": "0x337f9800" } }
Rule	Vaccine should be stored at a temperature between 2 and 8 Celsius degrees for maximum 5 days.
Transaction Receipt **(Storage Rule)**	**transaction hash:** 0xc5aa8f61103701da629ab3b5628e79ed8bf9729720d6df0e1ae1888f0e9711ec **from:** 0xD2796dE988975DD292e8aC981c4011B23E801DCd **to:** VaccineRegistry.registerTrackingRules(string, int32, int32, uint256) 0x536798D9D1f0507C1a8600d9A475d410a90D5A0A **transaction cost:** 200595 gas **input:** 0x4a2...26500 **decoded input** { "string rule": "medicalunit-storage-temperature", "int32 minValue": 2, "int32 maxValue": 8, "uint256 timeDelta": { "type": "BigNumber", "hex": "0x19bfcc00" } }

Similarly, the smart devices (freezers) that are responsible to store or transport the vaccine lots should be registered by associating one or more rules defined by the vaccine issuer to the freezer, so that the real-time data feeds can be checked against these rules automatically using the smart contracts (see Table 5).

**TABLE 5:** Blockchain Transactions for Registering the Freezing Devices

**Operation**	**Register Freezers**
Signing Address	0xD2796dE988975DD292e8aC981c4011B23E801DCd *(Vaccine Issuer)*
Transaction Receipt *(Transport Freezer)*	**transaction hash:** 0x1da71f023340fba695beeedf91ad447c7a41a726cf3296c3eb71e59fd360621e **from:** 0xD2796dE988975DD292e8aC981c4011B23E801DCd **to:** VaccineRegistry.registerFreezerAndRules(address,string) 0x536798D9D1f0507C1a8600d9A475d410a90D5A0A **transaction cost:** 46581 gas **input:** 0xe2d...00000 **decoded input** { "address freezer": "0xA53503C7901D09358F161eC8Ec8d442d0976B9cD", "string rule": "transport-temperature" }
Transaction Receipt *(Storage Freezer)*	**transaction hash:** 0x8d84c84d19995b9904e3a8bb9ebadea0fdd989f0388c92a3c303e1e8fde459bb
	**from:** 0xD2796dE988975DD292e8aC981c4011B23E801DCd
	**to:** VaccineRegistry.registerFreezerAndRules(address,string) 0x536798D9D1f0507C1a8600d9A475d410a90D5A0A
	**transaction cost:** 46701 gas
	**input:** 0xe2d...26500
	**decoded input** {
	"address freezer": "0xDDb54C6fbB74a5b638EF014f7435426C46424642",
	"string rule": "medicalunit-storage-temperature" }

At any point after the deployment of the smart contract, any beneficiary can subscribe to the waiting list for the vaccine. This is possible by issuing and signing a transaction on the chain. The address of the signing beneficiary (msg.sender) will be stored in the waiting list on-chain.

Upon subscription, the vaccine beneficiary must also provide a hash of his/her personal information (see Table 6).

**TABLE 6:** Blockchain Transactions for Registering the Vaccine Beneficiary

**Operation**	**Subscribe Beneficiary**
Signing Address	0xFfb4b11D94CFbbBA8665f7682D4d3B76261EAacC *(Beneficiary)*
Raw Personal Data	PI: 20-10563145-8 **Secret:** my-super-secret
Hashed-Personal Data	**Hash (PI):** 0xa3f6550e5420ddda304a6b22772eb70b48ada3c7eb14648e321bb65387c8cfab **Hash (Secret):** 0x820371900007448f4a8d909327870ece84168bf90f1de8dddc0b6c7473c44b40
Patient Hash	**Merkle Root:** 0xfe08609620228b43d9eb80125dfab7a1686e9c3cd7ea5326aa1c5abf7e689b87
Transaction Receipt	**transaction hash:** 0x76e5a604b3ca803e7738947dbc8616435d5fa410208e382a6d51b58d86b0374c **from:** 0xFfb4b11D94CFbbBA8665f7682D4d3B76261EAacC **to:** VaccineRegistry.registerPatient(bytes32) 0x536798D9D1f0507C1a8600d9A475d410a90D5A0A **transaction cost:** 84808 gas **input:** 0x8eb...89b87 **decoded input** { "bytes32 patientHash": "0xfe08609620228b43d9eb80125dfab7a1686e9c3cd7ea5326aa1c5abf7e689b87" }

*Vaccine tracking and administration*

Once the vaccine is ready the producer can register the vaccine lots on the blockchain system specifying the number of vaccine samples in a lot and the vaccine lot ID (see Table 7).

**TABLE 7:** Blockchain Transactions for Registering the Vaccine Batches

**Operation**	**Register Vaccine Lot**
Signing Address	0xD2796dE988975DD292e8aC981c4011B23E801DCd *(Vaccine Issuer)*
Transaction Receipt	**transaction hash:** 0x04ec1bc2f6a584810213b1521a515dc17e6f69ecda2e453ac1b554465cfa01b2
	**from:** 0xD2796dE988975DD292e8aC981c4011B23E801DCd
	**to:** VaccineRegistry.registerVaccineLot(bytes32, uint256) 0x536798D9D1f0507C1a8600d9A475d410a90D5A0A **transaction cost:** 64255 gas
	**input:** 0x0a1...000c8
	**decoded input** { "bytes32 vaccineLotId": “0xd7adb300b4c0d0f79bbb9195e3f9513b49caf8d14383062b2032d5656b13c5b5",
	"uint256 samples": { "type": "BigNumber", "hex": "0xc8" } }

Next, the vaccine lot is associated with one of the registered freezers. Each time a vaccine transfer is carried out on the distribution chain it will be marked on the blockchain chain by updating the freezer associated with the vaccine lot (see Table 8).

**TABLE 8:** Blockchain Transactions for Registering the Vaccine Lot

**Operation**	**Register Vaccine Lot for Transportation**
Signing Address	0xD2796dE988975DD292e8aC981c4011B23E801DCd *(Vaccine Issuer)*
Transaction Receipt	**transaction hash:** 0xf840c0653a1190a875825377afb7feacf130e7fc32d81bde887ae71ef388a7c9
	**from:** 0xD2796dE988975DD292e8aC981c4011B23E801DCd
	**to:** VaccineRegistry.updateVaccineFreezer(bytes32, address, address) 0x536798D9D1f0507C1a8600d9A475d410a90D5A0A
	**transaction cost:** 68106 gas
	**input:** 0xf63...6b9cd
	**decoded input** { "bytes32 vaccineLotId": "0xd7adb300b4c0d0f79bbb9195e3f9513b49caf8d14383062b2032d5656b13c5b5",
	"address freezerDeviceNew": "0xA53503C7901D09358F161eC8Ec8d442d0976B9cD", "address freezerDeviceOld": "0xA53503C7901D09358F161eC8Ec8d442d0976B9cD" }

During transportation, the freezer will register on the chain the values received from the associated sensors (in our case the values are received from temperature sensors). They will be registered in an immutable manner as transactions on the blockchain system allowing the future audit of the vaccine distribution conditions against the producer rules (see Table 9).

**TABLE 9:** Blockchain Transactions for Distribution Tracking

**Operation**	**Vaccine distribution tracking**
Signing Address	0xA53503C7901D09358F161eC8Ec8d442d0976B9cD *(Transport Freezer)*
Transaction Receipt	**transaction hash:** 0x0338487f1a35aa763bde543d39159e26875ab70742303cc583fe3ce638279998 **from:** 0xA53503C7901D09358F161eC8Ec8d442d0976B9cD to: VaccineRegistry.monitor (bytes32, string, int32) 0x536798D9D1f0507C1a8600d9A475d410a90D5A0A **transaction cost:** 160278 gas **input:** 0x524...00000 **decoded input** { "bytes32 vaccineLotId": "0xd7adb300b4c0d0f79bbb9195e3f9513b49caf8d14383062b2032d5656b13c5b5", "string rule": "transport-temperature", "int32 monitoredValue": -70}
Transaction Receipt	**transaction hash:** 0xc816e94acd01eebfd524f49b16e910f53910c91eda4445102c15a7653c0f8668 **from:** 0xA53503C7901D09358F161eC8Ec8d442d0976B9cD to: VaccineRegistry.monitor (bytes32, string, int32) 0x536798D9D1f0507C1a8600d9A475d410a90D5A0A **transaction cost:** 129212 gas **input:** 0x524...00000 **decoded input** { "bytes32 vaccineLotId": "0xd7adb300b4c0d0f79bbb9195e3f9513b49caf8d14383062b2032d5656b13c5b5", "string rule": "transport-temperature", "int32 monitoredValue": -55 } **decoded output** -
	logs [ { "from": 0x536798D9D1f0507C1a8600d9A475d410a90D5A0A", "topic": "0x285c0714a79fa669178437acfc343777469b86a755b376b8143ad3087951d959",
	"event": "BrokenRule", "args": { "0": "transport-temperature", "1": "0xd7adb300b4c0d0f79bbb9195e3f9513b49caf8d14383062b2032d5656b13c5b5", "2": "-55", "3": "1607426813",
	"rule": "transport-temperature", "vaccineLot": "0xd7adb300b4c0d0f79bbb9195e3f9513b49caf8d14383062b2032d5656b13c5b5",
	"value": "-55", "time": "1607426813" } } ]
	… more monitored values

After reaching the medical care center, the beneficiaries will be scheduled for having the vaccine administered. Any vaccine beneficiary once reaching the doctor's office will have to provide the personal identification information QR code. The doctor will scan the QR code which will offer information about the beneficiary and the transaction hash proving that the subscribing vaccination list transaction has been mined (Table 10).

**TABLE 10:** Beneficiary QR Code and Data Registered on Blockchain

**QR Code**	**Information stored**
<ifig/>	**PI:** 20-10563145-8 **Hash_Secret:** 0x820371900007448f4a8d909327870ece84168bf90f1de8dddc0b6c7473c44b40 **Contract:** 0x536798D9D1f0507C1a8600d9A475d410a90D5A0A **TX_Hash:** 0x76e5a604b3ca803e7738947dbc8616435d5fa410208e382a6d51b58d86b0374c

Using this information, the patient will be verified against the waiting list registered on chain (see Table 11).

**TABLE 11:** Checking Beneficiary Subscription to the Vaccine Waiting List

**Operation**	**Check Patient Subscription**
Signing Address	0xF3A1846C82c74EA5D5d32a9BB8759A8093C26eFa *(Doctor)*
Call (Calls are not mined they are only queries to check the state)	**from:** 0xF3A1846C82c74EA5D5d32a9BB8759A8093C26eFa
	**to:** VaccineRegistry.checkPatientRegistration(bytes32, bytes32, address)
	0x536798D9D1f0507C1a8600d9A475d410a90D5A0A
	**Input:** 0x615...eaacc
	**decoded input** {
	"bytes32 hashPI":
	"0xa3f6550e5420ddda304a6b22772eb70b48ada3c7eb14648e321bb65387c8cfab",
	"bytes32 hashSecret": "0x820371900007448f4a8d909327870ece84168bf90f1de8dddc0b6c7473c44b40",
	"address patient": "0xFfb4b11D94CFbbBA8665f7682D4d3B76261EAacC"}
	**decoded output** {"0": "bool: true"}

Once validated, the doctor will check the vaccine sample using its QR code associated, specifying the lot Id and the address of the blockchain smart contract where the tracking information is registered (see Table 12).

**TABLE 12:** Vaccine Identification QR Code

**QR Code**	**Information stored**
<ifig/>	V_ID
	0xd7adb300b4c0d0f79bbb9195e3f9513b49caf8d14383062b2032d5656b13c5b5
	**Contract:**
	0x536798D9D1f0507C1a8600d9A475d410a90D5A0A

Using this information, the patient can verify the tracking information and whether the vaccine lot was correctly transported (see Table 13).

**TABLE 13:** Check Vaccine Distribution Tracking Information

**Operation**	**Check Vaccine History**
Signing Address	0xFfb4b11D94CFbbBA8665f7682D4d3B76261EAacC (*Beneficiary*)
Call (Calls are not mined they are only queries to check the state)	**From:** 0xFfb4b11D94CFbbBA8665f7682D4d3B76261EAacC
	**To:** VaccineRegistry.checkVaccineLotHistory(bytes32)
	0x536798D9D1f0507C1a8600d9A475d410a90D5A0A
	**Input:** 0xfb7...3c5b5
	**decoded input** { "bytes32 vaccineLotId": "0xd7adb300b4c0d0f79bbb9195e3f9513b49caf8d14383062b2032d5656b13c5b5" }
	**decoded output** { "0": "tuple (address, string,int32,uint256,bool)[]:
	0xA53503C7901D09358F161eC8Ec8d442d0976B9cD, transport-temperature, -70, 1607426735, true, 0xA53503C7901D09358F161eC8Ec8d442d0976B9cD,
	transport-temperature, -55, 1607426813, false, 0xDDb54C6fbB74a5b638EF014f7435426C46424642,
	medicalunit-storage-temperature, 5, 1607427059, true, 0xDDb54C6fbB74a5b638EF014f7435426C46424642,
	medicalunit-storage-temperature, 10, 1607427155, false"}

Once the vaccine is administered, a multi-signature is required on the chain for updating the vaccine lot size and marking one vaccine sample as administered. The signatures are expected from both the receiving beneficiary and the doctor which has administrated the vaccine (see Table 14).

**TABLE 14:** Signing the Acknowledgement of Vaccine Administration

**Operation**	**Signing**
Signing Address	0xFfb4b11D94CFbbBA8665f7682D4d3B76261EAacC (*Beneficiary*)
Transaction Receipt	**transaction hash:** 0x24813e343ccb5f4b5916367c5337385c494ff3e576a5e1ffbcd5c9ee1f424508
	**from:** 0xFfb4b11D94CFbbBA8665f7682D4d3B76261EAacC
	to: VaccineRegistry.signAdministeredVaccine(bytes32, bytes32) 0x536798D9D1f0507C1a8600d9A475d410a90D5A0A
	**transaction cost:** 49401 gas
	**input:** 0x4df...8cfab
	**decoded input** { "bytes32 vaccineLotId": "0xd7adb300b4c0d0f79bbb9195e3f9513b49caf8d14383062b2032d5656b13c5b5", "bytes32 hashPI": "0xa3f6550e5420ddda304a6b22772eb70b48ada3c7eb14648e321bb65387c8cfab" }
Signing Address	0xF3A1846C82c74EA5D5d32a9BB8759A8093C26eFa (*Doctor*)
Transaction Receipt	**transaction hash:** 0xf8d21694b2008cd123fa89899ed5b3a330df4460c53f25d3d15e8b1cdef76d4e
	**from:** 0xF3A1846C82c74EA5D5d32a9BB8759A8093C26eFa
	**to:** VaccineRegistry.signAdministeredVaccine (bytes32,bytes32) 0x536798D9D1f0507C1a8600d9A475d410a90D5A0A
	**transaction cost:** 74528 gas
	**input:** 0x4df...8cfab
	**decoded input** { "bytes32 vaccineLotId": "0xd7adb300b4c0d0f79bbb9195e3f9513b49caf8d14383062b2032d5656b13c5b5", "bytes32 hashPI": "0xa3f6550e5420ddda304a6b22772eb70b48ada3c7eb14648e321bb65387c8cfab" }

Once both signatures are received, the vaccine is considered successfully administrated and the vaccine lot size decreases by one. This can be verified on-chain by any participant. After receiving the vaccine, the patient can register voluntarily any side effect he is feeling (see Table 15).

**TABLE 15:** Potential Side Effects Reporting

**Operation**	**Register Side effect**
Signing Address	0xFfb4b11D94CFbbBA8665f7682D4d3B76261EAacC (*Beneficiary*)
Transaction Receipt	**transaction hash:**0x7ed30d2cd822d854bc36ba666c657e5029e7f6d72c1c67202aabff459a658a13
	**from:** 0xFfb4b11D94CFbbBA8665f7682D4d3B76261EAacC
	**to:** VaccineRegistry.registerSideEffect(bytes32, bytes32, bytes32, string) 0x536798D9D1f0507C1a8600d9A475d410a90D5A0A
	**transaction cost:** 48073 gas
	**input:** 0x56f...00000
	**decoded input** { "bytes32 hashPI": "0xa3f6550e5420ddda304a6b22772eb70b48ada3c7eb14648e321bb65387c8cfab", "bytes32 hashSecret": "0x820371900007448f4a8d909327870ece84168bf90f1de8dddc0b6c7473c44b40", "bytes32 vaccineLotId": "0xd7adb300b4c0d0f79bbb9195e3f9513b49caf8d14383062b2032d5656b13c5b5", "string sideEffect": "Dizziness, Nausea" }

### B. System Throughput and Cost

The integration of monitored data feed of vaccine distribution condition directly on blockchain may feature high costs associated with the cumulated blockchain transactions and poor scalability determined by the block mining periodicity and gas limit imposed per block. To deal with these issues we have integrated a pre-processing step at the edge level (physical device level) that is responsible to receive all the data from one sensor deployed on the vaccine distribution network and determine for an interval the most significant values are registered on the chain. In this case, the most significant values are the lowest and the highest temperature values registered in that interval. This solution had been presented in detail in one of our previously published research [45].

For scalability experiments, we have considered the setup and restrictions in terms of gas limits (approximately 12 000 000 per block) and mining periodicity (i.e., 15 seconds) of the public Ethereum main network. In our blockchain-based system case, we obtain a transaction cost of approximately 140 000 gas for registering the monitored temperature of the freezing devices concerning vaccine safe delivery rules and transaction throughput of approximately 85 transactions per block.

**Figure 9.**
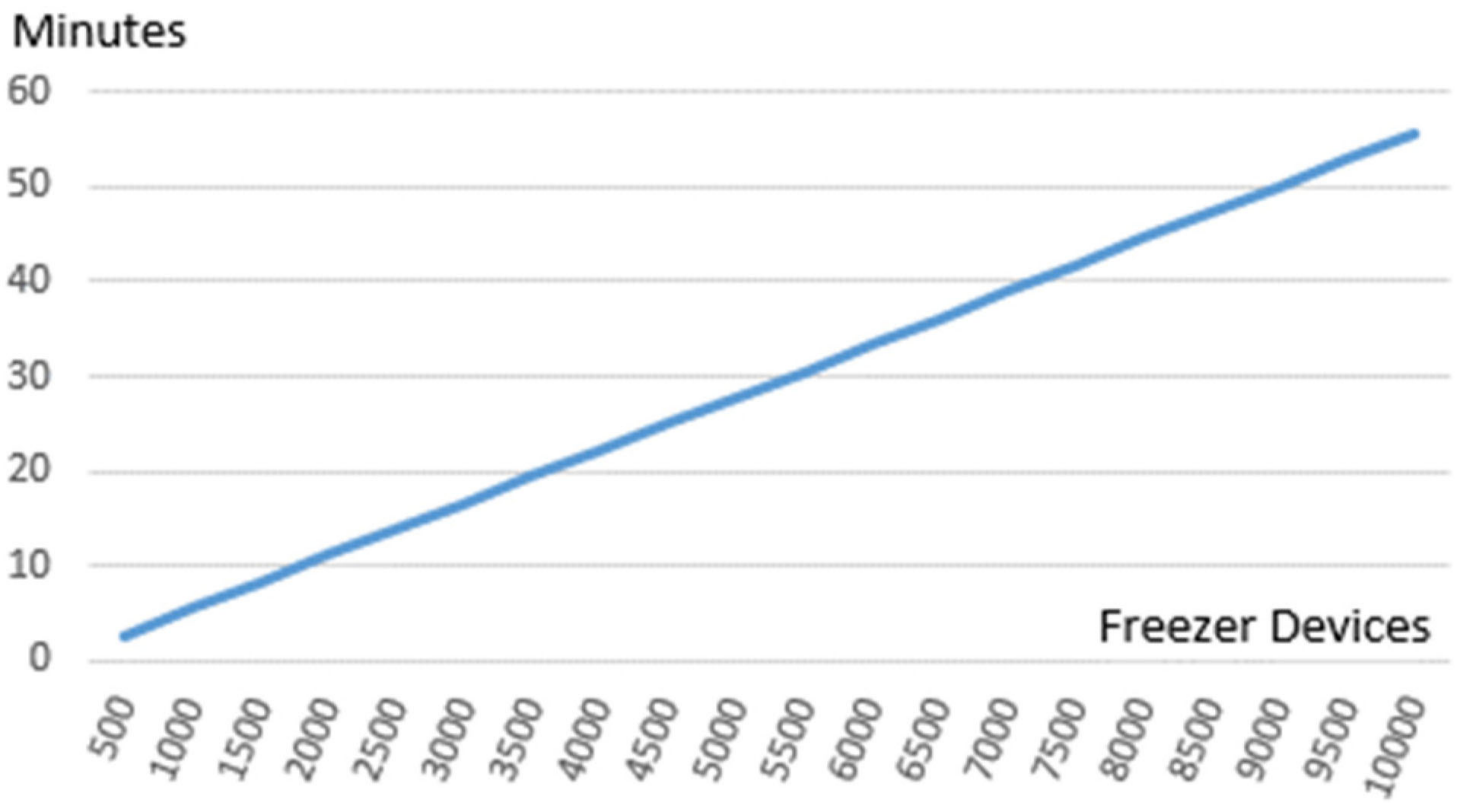
Mining time for vaccine distribution temperature tracking transactions.

The transaction mining results are presented in Fig. 9 considering an interval of one hour in which each device will have to store two temperature monitoring transactions (one for the minimum registered value and one for the maximum registered value). Up to 10 000 freezers that are exclusively signing transactions on the blockchain network can be efficiently managed without creating a bottleneck. Being a linear relationship one can easily change the interval for reporting monitored temperature transactions to accommodate more transportation devices.

To analyze the system costs in terms of public blockchain gas consumption we have considered the real-life scenario based on the activity reported vaccination campaign in Romania [64], [65]. On the 11^th^ of January, 150.150 Pfizer vaccine doses have arrived in Romania. There are 195 vials per package, thus 770 packages (i.e., lots) of vaccines have been delivered in the country that day. On the 14^th^ of January there were 309 vaccination centers deployed across the country, and on the 15^th^ of January vaccines have been administered to 16057 beneficiaries. From this group 68 have reported side effects: 24 mild and local side effects and 44 generalized side effects like fever and headaches. For one week (15--21^st^ January), 491342 persons have registered in the national platforms for vaccination, leading to a mean of 70192 per day.

Table 16 reports the gas consumption per system operation and the number of operations required for one day considering the defined scenario. For our testing setup, we have considered that the previously reported number for system activities are registered uniformly during the hours of the day.

**Table 16:** Operation Costs and Frequency

Operation	Gas/Operation	No. operations
Register Vaccine lot	64255	770
Register Lot for Storage	68106	770
Subscribe Beneficiary	84808	70192
Sign Administration & Update Lot	49401 + 74528 = 123929	16057
Side effects	48073	68
Monitor	∼140000	18480

In our evaluation, we considered that on the same day of the week the vaccines are delivered, and the lots also are registered in the system. Even if the registration operations would normally precede the delivery ones with at least one day, the assumption made allowed us to determine the aggregated gas consumption results. The blockchain system operations of freezers monitoring, beneficiary registration, and side effects reporting are distributed evenly throughout the entire day. The vaccine administration, the registration of the vaccine lots, and the arrival of the vaccine lot in the medical storage unit are blockchain system operations performed by specialized personnel during a 12-hour program (from 8 in the morning until 20 in the afternoon).

**Figure 10.**
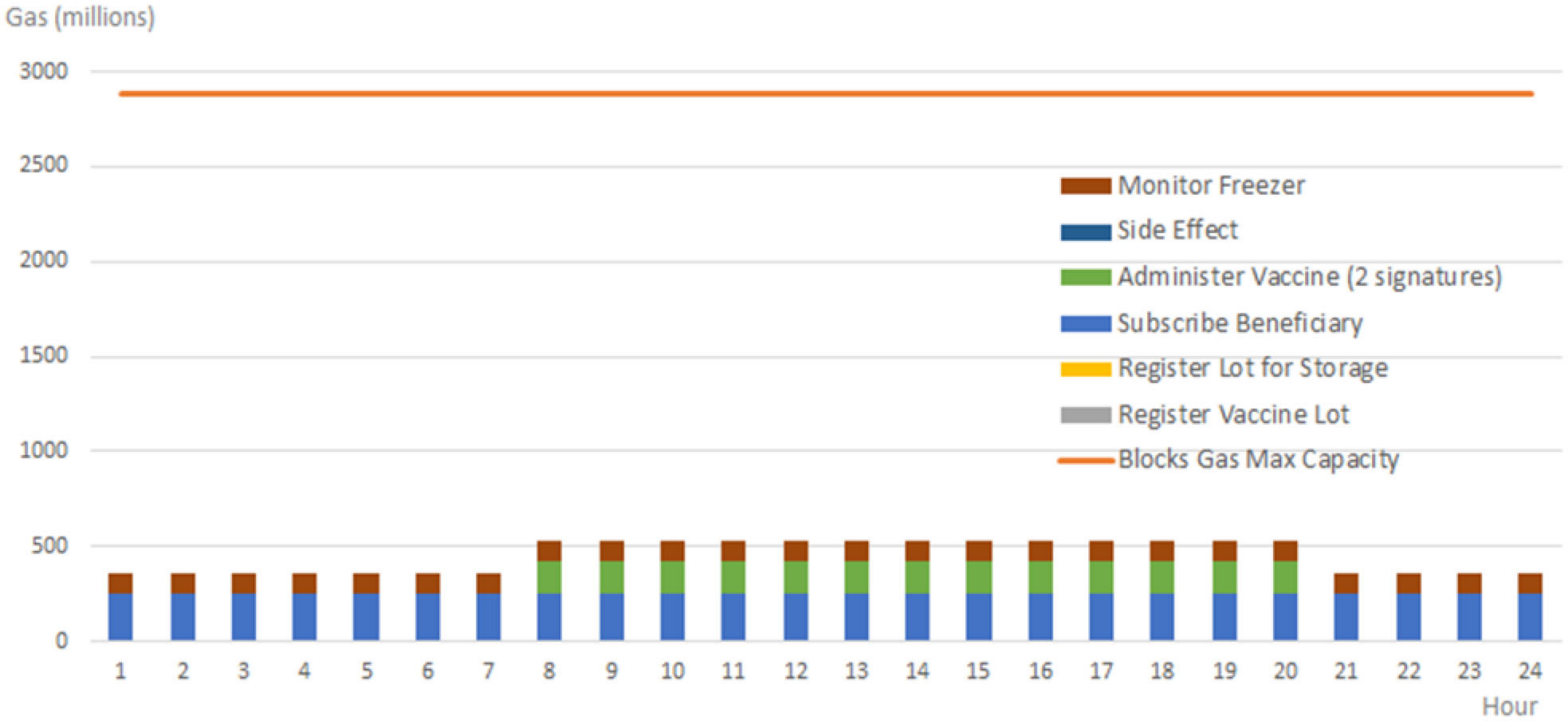
Blockchain system cost in terms of transactions gas consumption during a vaccination day.

The capacity of the blockchain to process transactions is given by the maximum amount of gas that can be consumed by the transactions mined in a block. Specifically, for our setup, we considered the block capacity of 12 000 000 gas, corresponding to the gas limit imposed by the Ethereum public chain at the time of writing. As a result, a gas limit of 2880 million gas is determined per hour, considering a mining rate of a block at 15 seconds. Fig. 10 shows the gas consumption corresponding to the system operations in a day for our scenario. Even though we have considered a higher number of operations than normal the gas consumed is at least 6 times lower than the maximum capacity. Thus, many more vaccines administration to beneficiaries could be managed by our system in a day.

Besides the cost, in terms of gas consumption, and transaction throughput limitations another challenge in a real work setting is related to data privacy on one hand and the system auditability and tracking on the other hand. For example, to fulfil the General Data Protection Regulation (GDPR) requirements [66] in Europe techniques like zero-knowledge proof [67] may need to be considered. Also, the GDPR can make difficult the integration of the blockchain system with other public health and location services. This might be needed some time for example for integrating the beneficiary history for allergies prior to vaccination to be used later for self-reporting of side effects and audit.

## VI. Conclusion

In this paper we presented a blockchain-based system for transparent tracing of COVID-19 vaccine registration, storage and delivery and side effects self-reporting. Blockchain is used to offer data immutability, transparency, and correctness of beneficiary registration for vaccination, avoiding identity thefts and impersonations. The tracking and monitoring of vaccine distribution against the producer defined rules for safe manipulation is done using decentralized smart contracts. Also, a blockchain solution is proposed for vaccine administration and transparent and tamper-proof self-reporting of side effects, person identification, and vaccine association.

The results provided for an Ethereum based implementation show the feasibility of our proposed solution in terms of transaction throughput and cost in terms of gas consumption considering as reference scenario the immunization campaign from our country. The proposed system manages to successfully address all relevant aspects we had identified for a successful monitoring campaign: (i) increase the efficiency and transparency of COVID-19 vaccine distribution assuring the traceability and the rigorous audit of the storage and delivery conditions (ii) assure the transparency and correctness in the registration and management of the waiting list for immunization and (iii) provide a transparent and public reporting system of potential side effects.
